# Genotoxic Agents Promote the Nuclear Accumulation of Annexin A2: Role of Annexin A2 in Mitigating DNA Damage

**DOI:** 10.1371/journal.pone.0050591

**Published:** 2012-11-30

**Authors:** Patricia A. Madureira, Richard Hill, Patrick W. K. Lee, David M. Waisman

**Affiliations:** 1 Departments of Biochemistry and Molecular Biology and Pathology, Dalhousie University, Halifax, Nova Scotia, Canada; 2 Department of Microbiology and Immunology, Dalhousie University, Halifax, Nova Scotia, Canada; 3 Centre for Molecular and Structural Biomedicine, University of Algarve, Campus de Gambelas, Faro, Portugal; St. Georges University of London, United Kingdom

## Abstract

Annexin A2 is an abundant cellular protein that is mainly localized in the cytoplasm and plasma membrane, however a small population has been found in the nucleus, suggesting a nuclear function for the protein. Annexin A2 possesses a nuclear export sequence (NES) and inhibition of the NES is sufficient to cause nuclear accumulation. Here we show that annexin A2 accumulates in the nucleus in response to genotoxic agents including gamma-radiation, UV radiation, etoposide and chromium VI and that this event is mediated by the nuclear export sequence of annexin A2. Nuclear accumulation of annexin A2 is blocked by the antioxidant agent N-acetyl cysteine (NAC) and stimulated by hydrogen peroxide (H_2_O_2_), suggesting that this is a reactive oxygen species dependent event. In response to genotoxic agents, cells depleted of annexin A2 show enhanced phospho-histone H2AX and p53 levels, increased numbers of p53-binding protein 1 nuclear foci and increased levels of nuclear 8-oxo-2′-deoxyguanine, suggesting that annexin A2 plays a role in protecting DNA from damage. This is the first report showing the nuclear translocation of annexin A2 in response to genotoxic agents and its role in mitigating DNA damage.

## Introduction

Annexins are a structurally related family of calcium and phospholipid-binding proteins that are involved in the regulation of a broad range of molecular and cellular processes [1], [2]. Annexins bind to anionic phospholipids in a calcium (Ca^2+^)–dependent manner. All annexins share a conserved domain of 4 repeat sequences of approximately 70 residues long composed of 5 α-helices containing several Ca^2+^ binding sites [3]–[5]. Annexin A2 is present in cells in two forms, as a monomer or a heterotetramer (AIIt). The heterotetramer (AIIt) consists of two molecules of annexin A2 linked together by a dimer of the protein S100A10 [3], [6], [7]. The N-terminal domain of annexin A2 contains the binding site for S100A10 [8], a reactive cysteine residue [9], [10], phosphorylation sites [11], [12] and a nuclear export signal (NES) [13], while the C-terminal domain of annexin A2 contains binding sites for F-actin [14], phospholipid [4], [5], [15], fibrin [16] and heparin [17].

Annexin A2 is primarily localized in the cytoplasm and plasma membrane [18] with a smaller but significant population in the nucleus [13], [19], [20]. Although the role of cytoplasmic and membrane associated annexin A2 has been extensively studied, the role of nuclear annexin A2 is unclear. One study reported that 15% of total annexin A2 was present in the nucleus of fibroblasts and was released by RNase A [19], consistent with the identification of annexin A2 as an RNA-binding protein [21]. Nuclear annexin A2 has also been suggested to play a role as part of a primer recognition protein complex that enhances DNA polymerase α activity *in vitro* [22].

Annexin A2 has a nuclear export sequence that prevents the nuclear accumulation of the protein. Mutations of lysines 10 and 12 within the NES of annexin A2 inactivates this motif and results in nuclear accumulation. Thus, it has been proposed that the nuclear accumulation of annexin A2 occurs only after inactivation of its NES. The mechanism by which annexin A2 enters the nucleus is unclear as this protein does not possess a traditional nuclear import sequence [13]. Annexin A2 phosphorylation has also been suggested to play a role in annexin A2 nuclear accumulation and overall localization; however these reports are contradictory. For example, phosphorylation of both Ser 11 and 25 is reported to block the nuclear accumulation of annexin A2 [23], while phosphorylation of Ser 25, has been suggested to be necessary for its nuclear accumulation [12]. In contrast, phosphorylation of Tyr 23 of annexin A2 may activate its nuclear translocation [13], activate its association with endosomes [24], [25] and stimulate its translocation to the cell surface [26]. Although the importance of the NES in regulating the nuclear accumulation of annexin A2 has been established, the cellular processes that trigger the nuclear accumulation of annexin A2 are still unknown.

Previous work from our laboratory has shown that annexin A2 is an important redox regulatory protein, particularly in cells undergoing oxidative stress. Depletion of annexin A2 resulted in increased protein oxidation in cells challenged with mild oxidative stress, suggesting that annexin A2 protects cellular proteins from oxidation [10]. In the present report, we examined the cellular distribution of annexin A2 in cells subjected to genotoxic stress. We observed a rapid nuclear accumulation of annexin A2 in response to DNA damaging agents including gamma-radiation, UV radiation, chromium VI and the chemotherapeutic agent, etoposide. Interestingly, the nuclear accumulation of annexin A2 in response to genotoxic agents was blocked by the antioxidant N-acetyl cysteine (NAC), suggesting that reactive oxygen species (ROS) produced by the genotoxic agents induced the nuclear accumulation of the protein. Consistent with this hypothesis, we observed that annexin A2 rapidly accumulated in the nucleus of cells subjected to oxidative stress caused by hydrogen peroxide (H_2_O_2_) and that this nuclear accumulation was regulated by the NES of annexin A2. Finally, we showed that annexin A2 depleted cells are more sensitive to DNA damage, compared to control cells. This is the first report describing the nuclear translocation of annexin A2 in response to genotoxic agents and its role in mitigating DNA damage.

## Materials and Methods

### Cell Culture, Transfections and Cell Lines

293T, MCF7 and A549 cells were obtained from ATCC and maintained in Dulbecco’s modified Eagle’s medium (Invitrogen) supplemented with 10% fetal bovine serum (FBS) and 100 U/ml of penicillin/streptomycin, in a humidified incubator in an atmosphere of 5% CO_2_ at 37°C. TIME endothelial cells were a kind gift from Dr McMahon [27] and maintained in EGM2 medium (Lonza) supplemented with 2% FBS and 100 U/ml of penicillin/streptomycin, in a humidified incubator in an atmosphere of 5% CO_2_ at 37°C. 293T cells in 6 well plates were transfected with 1 µg of the GFP plasmids described in File S1 using 3 µl of the lipofectamine 2000 transfection reagent according to the manufacturers’ instructions. Annexin A2 depleted cell lines were obtained by transfection of Phoenix packaging cells with 4 µg of the pSUPER-retro plasmids described in File S1 using 12 µl of the lipofectamine 2000 transfection reagent according to the manufacturers’ instructions. 48 hours after transfection the target cells were infected with Phoenix supernatants and selected with 2 µg/ml of puromicin.

### Plasmids

Plasmids are detailed in File S1.

### Antibodies

The following antibodies were used for western blot analysis: annexin A2 antibody #610069 (BD Transduction laboratories), S100A10 antibody #610071 (BD Transduction laboratories), actin antibody (AC-40) #A3853 (SIGMA), p53 antibody (DO-1) #sc-126 (SCBT), β-tubulin antibody (H-235) # sc-9104 (SCBT), Lamin A/C antibody (N-18) #sc-6215 (SCBT), nucleolin antibody (D-6) #sc-17826 (SCBT), JunD antibody #sc-44 (SCBT), CD146 antibody #sc-81614 (SCBT). Antibodies used for immunocytochemistry were the following: annexin A2 antibody #ab41803-100 (AbCam), S100A10 antibody #610071 (BD Transduction laboratories), hnRNP A2/B1 antibody #sc-53531 (SCBT), anti-rabbit 488 Alexa Fluor #A11008 (Molecular Probes) and anti-mouse 546 Alexa Fluor #A11003 (Molecular Probes).

### Immunoprecipitation Assays

Cells were washed with PBS. For annexin A2 and S100A10 co-immunoprecipitations, cells were lysed with NP-40 lysis buffer (20 mM Tris pH 7.4, 1% NP-40, 150 mM NaCl, 2 mM EGTA, protease inhibitors, 1 mM NaVO_4_, 10 mM NaF) for 10 minutes on ice. Cell lysates were pre-cleared for 1 h with protein G-Sepharose, incubated with specific antibodies for 1 h and then with 50% slurry of protein G-Sepharose for 1 h. Beads were washed five times with 500 µl of lysis buffer, resuspended with 25 µl 2X SDS-PAGE loading buffer, boiled for 5 minutes, subjected to SDS-PAGE and analysed by western blot. The following antibodies were used for immunoprecipitation studies: annexin A2: D1/274.5 mouse monoclonal (made in house), S100A10: #610071 (BD Transduction laboratories), JunD: #sc-44 (SCBT) and Trx: sc-20146 (SCBT).

### Western Blot Analysis

For western blot analysis 20 µg of cell lysates from total cell extracts or equal ratios of each fraction (corresponding to the same percentage of protein from each fraction) for cellular fractionation experiments, were subjected to SDS-PAGE, transferred onto a nitrocellulose membrane, incubated with appropriate antibodies and visualized using a Licor Odyssey scanner (Li-cor Biosciences).

### Sub-cellular Fractionation

Typically cells were mock treated, or treated with 1.5 J/m^2^ of UV-A (365 nm) using a UV stratalinker (Stratagene), 10 Gy of Gamma-irradiation using a Gammacell 3000 Elan irradiator (MDS Nordion), 8.5 µM etoposide or 20 µM Cr(VI). Cells were washed with PBS and scraped with buffer A (50 mM Hepes pH 7.4, 0.1% Triton-X100, 10 mM NaCl, 20% glycerol, 1 mM EDTA, 2 mM EGTA, 2 mM MgCl_2_, 1 mM DTT, 1 mM NaVO_4_, 10 mM NaF and protease inhibitors). Lysates were incubated for 20 minutes at 4°C and centrifuged at 500 g for 3 minutes. Supernatants containing the non-nuclear fractions were stored. The pellets were resuspended with buffer B (50 mM Hepes pH 7.4, 0.5% Triton-X100, 400 mM NaCl, 1 mM EDTA, 2 mM EGTA, 1 mM MgCl_2_, 1 mM DTT, 1 mM NaVO_4_, 10 mM NaF and protease inhibitors), incubated for 20 minutes at 4°C and centrifuged at 12000 g for 15 minutes. The supernatants containing the nuclear fractions were recovered. Identical ratios, representing the same percentage of each subcellular fraction (nuclear and non nuclear fractions) were subjected to SDS-PAGE followed by western blotting, in order to represent the cellular ratio of distribution of annexin A2 in each compartment. Typically, 15 µg of non nuclear fraction and 2–2.5 µg of nuclear fraction were loaded. The same amount of protein was loaded within each fraction. The nuclear versus non nuclear ratio varied slightly for different cell lines, but it was usually approximately 1∶6–7 for all cell lines used.

### Nuclear/Cytoplasmic/Membrane/Cytoskeleton Fractionation

For multi-compartmental fractionation of cells we used the CNMS compartment protein extraction kit (Biochain Institute) according to the manufacturer’s instructions. Identical ratios, representing the same percentage of each subcellular fraction were subjected to SDS-PAGE followed by western blotting, in order to represent the cellular ratio of distribution of annexin A2 in the different cellular compartments.

### Immunocytochemistry

Cells were fixed with 2% formaldehyde for 30 minutes, permeabilized with 0.1% Triton X-100 for 15 minutes and blocked with IgG for 1 hour. Primary antibodies were incubated for 1 hour individually, followed by incubation with secondary antibodies for 1 hour. Cells were visualized by confocal microscopy using a Zeiss LSM 510 META - Laser Scanning Confocal Microscope (Carl Zeiss Inc.).

### Fluorescence Microscopy

293T cells transfected with different GFP expression plasmids for 48 hours were visualized under a fluorescence microscope using a GFP filter and pictures were taken (non treated cells), after what these cells were treated with H_2_O_2_ for 30 minutes and pictures were taken (H_2_O_2_ treated cells). Fluorescent cells were visualized using a Research Macro Zoom System MVX10 fluorescence microscope (Olympus).

### 53BP1 Foci Staining and Scoring

TIME annexin A2 shRNA2 and scramble (control) cells were plated on microscope cover slips in EGM2-10% FCS and incubated at 37°C for 24 h. Cells were then mock treated or treated with either IR or H_2_O_2_ at the indicated dosages for 6 hours. Cover slips were washed once with PBS and then fixed and permeabilized for 30 minutes using −20°C cold methanol. Cover slips were washed three times with PBS. Cells were incubated in a humidified chamber at 37°C for 30 minutes with goat serum (Gibco, USA) diluted 1∶30.The serum was removed and the slides were incubated with anti-53BP1 antibody (SCBT, USA) at 1∶500 for 1 hour. After washing three times with PBS for 10 minutes at room temperature, cover slips were incubated with the appropriate Cy2-conjugated secondary antibody (Molecular Probes, USA) in a humidified chamber at 37°C for 30 minutes. After washing as described above, cover slips were mounted on glass slide using DAPI Vector mounting solution (Vector Laboratories, UK) and analyzed. 53BP1 foci were visualized at 100× magnification using a Zeiss LSM 510 META - Laser Scanning confocal microscope. For each condition the number of 53BP1 foci was scored within 100 nuclei from triplicate cover slips. For each 53BP1 foci scored, the diameter was measured using the LSM imaging software Zen 2009 light edition. Once scored and measured, the data was plotted using Graphpad Prism. Statistical significance was assessed by two-way ANOVA or the two-tailed Students t-test. Statistical significance was defined as P<0.05. Results are expressed as the mean ± StDev.

### OxyDNA Assay

Oxidative DNA damage was accessed with the Argutus Medical OxyDNA Test Kit (Argutus Medical) according to the manufacturers’ instructions. Briefly, cells were either not treated or treated with 10 Gy IR for 6 hours. Cells were washed twice with PBS and fixed with 2% formaldehyde/PBS for 15 minutes at 4°C. Cells were washed with PBS and permeabilized with 70% ethanol/PBS overnight at −20°C. Cells were then washed with TBS-T, resuspended in 100 µl of 8-oxoguanine binding protein-FITC conjugate dilution (1∶10) in TBS-T and incubated for 2 hours at room temperature. Cells were washed with TBS-T and analysed by FACS. Statistical significance was assessed by the two-tailed Students t-test, N = 6. Statistical significance was defined as *P<0.05, **P<0.002, *** P<0001. Results are expressed as the mean ± StDev.

## Results

### Annexin A2 Accumulates in the Nucleus in Response to Genotoxic Agents in a ROS Dependent Manner

**Figure 1:**
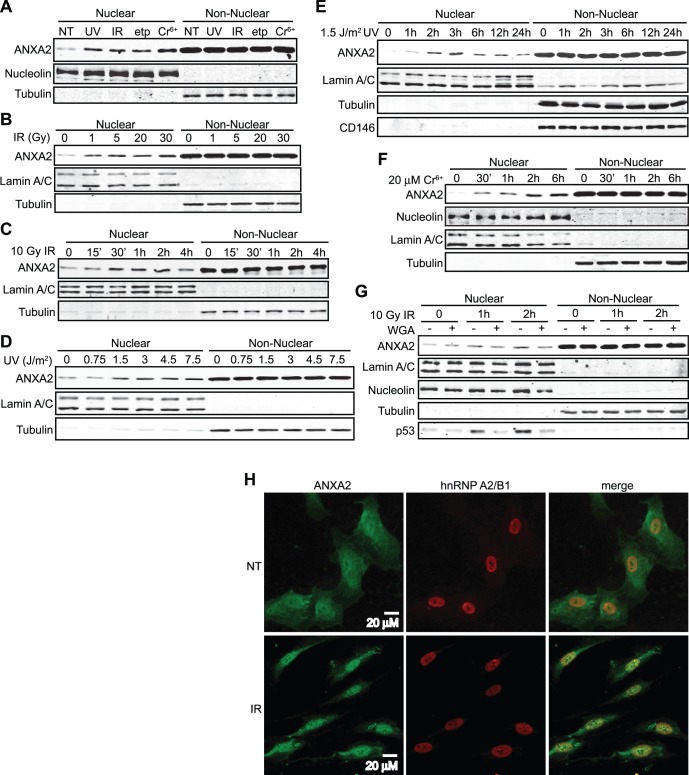
Annexin A2 translocates into the nucleus in response to genotoxic agents. TIME cells were not treated (NT) or treated with: (A) 1.5 J/m^2^ UV-A (365 nm) (UV), 10 Gy Gamma-radiation (IR), 8.5 µM etoposide (etp) or 20 µM chromium VI (Cr6^+^) for 2 hours; (B) with the indicated doses of Gamma-radiation (IR) for 2 hours; (C) with 10 Gy of IR for the times indicated; (D) with the indicated doses of UV-A radiation for 2 hours; (E) with 1.5 J/m^2^ UV-A (365 nm) for the times indicated; (F) with 20 µM chromium VI (Cr^6+^) for the times indicated; (G) with 10 Gy of IR in the absence or presence of Wheat Germ Aglutinin (WGA) for the times indicated. (A–G) Nuclear and Non Nuclear fractions were prepared and identical ratios, representing the same percentage of each subcellular fraction were subjected to SDS-PAGE followed by western blotting with the antibodies indicated. Protein markers for the sub-cellular fractions include: nucleolin (nucleus); lamin A/C (nuclear membrane); tubulin (cytoplasm); CD146 (plasma membrane). (H) TIME cells were either not treated (NT) or treated with 10 Gy IR for 1 hour. Cells were subjected to immunocytochemistry analysis with the antibodies indicated and visualized by confocal microscopy. Scale bar is 20 µM.

Although annexin A2 is present in the nucleus; its role in this cellular compartment is unclear. Previous work from our laboratory has shown that annexin A2 is a redox regulatory protein that protects cellular proteins by interacting with ROS such as H_2_O_2_ [10]. With this work we further investigated the cellular localization of annexin A2 in response to genotoxic agents that produce ROS. In order to investigate this, we treated telomerase immortalized microvascular endothelial (TIME) cells with genotoxic agents such as ultra-violet (UV) radiation, gamma-radiation (IR), chromium VI (Cr^6+^) [28] and the chemotherapeutic agent, etoposide and observed that annexin A2 rapidly accumulated in the nucleus (Figure 1A). Significant nuclear accumulation of annexin A2 occurred by 15 minutes after treatment with 10 Gy of IR and peaked by 30 minutes to 2 hours after treatment (Figure 1C). In contrast, nuclear annexin A2 levels peaked by 3 hours after treatment with 1.5 J/m^2^ of ultra-violet radiation (Figure 1E) and about 2 hours after treatment with 20 µM chromium VI (Figure 1F). The amount of nuclear annexin A2 was also dependent on the dose of the genotoxic agent (Figure 1B, D).

Although the results of our subcellular fractionation studies suggested that annexin A2 accumulated in the nucleus in response to genotoxic agents, we could not rule out the possibility that annexin A2 did not enter the nucleus, but was associated with the cytoplasmic surface of the nuclear membrane. However, we observed that the nuclear accumulation of annexin A2 in response to genotoxic agents was blocked by the nuclear influx inhibitor, wheat germ agglutinin (WGA) (Figure 1G) [29], [30]. Furthermore, immunofluorescence microscopic analysis demonstrated the nuclear co-localization of annexin A2 with the nuclear marker, hnRNP A2/B1 in response to IR (Figure 1H and S1A, B). This result suggested that the nuclear accumulation of annexin A2 observed in our subcellular fractionation studies was due to the entry of annexin A2 into the nucleus. The nuclear accumulation of annexin A2 induced by genotoxic agents was also observed in a number of different cell lines including HUVEC, A549, MCF7 and HCT 116 cells (Figure S2A, B, C, D).

**Figure 2:**
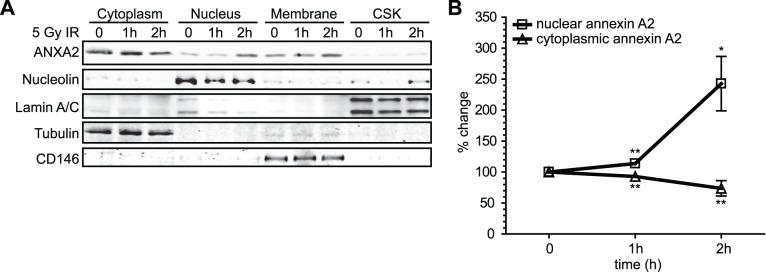
Annexin A2 accumulates in the nucleus and decreases in the cytoplasm in response to genotoxic agents. (A) TIME cells were not treated (NT) or treated with 5 Gy IR for the times indicated. Cells were fractionated into nuclear, cytoplasmic, membrane and cytoskeletal (CSK) fractions. Identical ratios of the various protein fractions, representing the same percentage of each subcellular fraction, were subjected to SDS-PAGE followed by western blotting with the antibodies indicated. Protein markers for the sub-cellular fractions include: nucleolin (nucleus/cytoskeleton); lamin A/C (nuclear membrane and cytoskeleton); tubulin (cytoplasm); CD146 (plasma membrane). (B) Quantification of the change in annexin A2 protein expression in the nucleus and cytoplasm before and after IR treatment using the Licor Odyssey software. These results represent the average of 4 independent experiments (N = 4). Statistical significance was defined as *P<0.05, **P<0.002, *** P<0.0001. Results are expressed as the mean ± StDev.

We also analyzed the subcellular distribution of annexin A2 using a multi-compartmental cell fractionation system. We observed that the nuclear accumulation of annexin A2 in response to genotoxic agents was concomitant with a loss in cytoplasmic annexin A2, while the annexin A2 levels in the membrane and cytoskeleton fractions did not change significantly (Figure 2A). Densitometric analysis suggested that in non treated cells about 10% of total cellular annexin A2 was nuclear, consistent with previously published data [19]. At 2 hours after treatment with 5 Gy of IR we observed approximately a 3 fold increase in the amount of nuclear annexin A2 (30% of total cellular protein) (Figure 2B).

**Figure 3:**
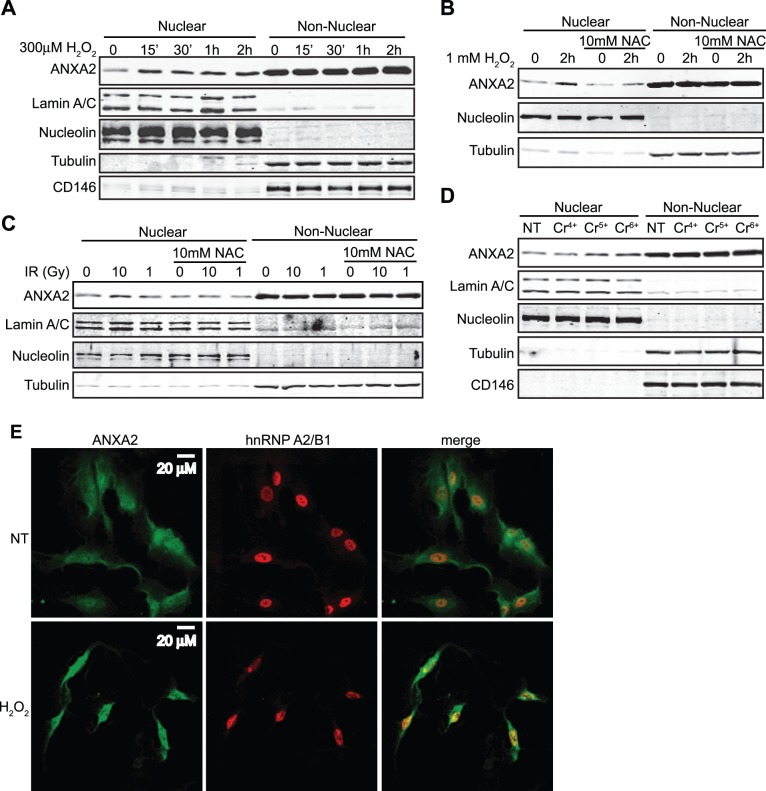
Annexin A2 nuclear accumulation is dependent of ROS. TIME cells were treated with (A) 300 µM H_2_O_2_ for the times indicated; (B) 1 mM H_2_O_2_ in the absence or presence of 10 mM NAC for 2 hours; (C) 1 Gy or 10 Gy of IR in the absence or presence of 10 mM NAC for 2 hours; (D) 20 µM Cr^4+^, Cr^5+^ or Cr^6+^ as indicated, for 2 hours. (A-D) Nuclear and non-nuclear fractions were prepared. Identical ratios of the various protein fractions were subjected to SDS-PAGE followed by western blotting with the antibodies indicated. (E) TIME cells were either not treated (NT) or treated with 0.5 mM H_2_O_2_ for 30 minutes. Cells were subjected to immunocytochemistry analysis with the antibodies indicated and visualized by confocal microscopy. Scale bar is 20 µM.

The induction of nuclear accumulation of annexin A2 by a diverse group of genotoxic agents presented the possibility that these agents acted by a common mechanism. Since IR, UV, etoposide and Cr^6+^ are known to induce ROS; we investigated the possibility that the nuclear accumulation of annexin A2 was stimulated by oxidative stress. Accordingly, cells were incubated with H_2_O_2_ and the nuclear accumulation of annexin A2 was analyzed by cell fractionation followed by western blotting. We observed that in response to 300 µM H_2_O_2_, annexin A2 rapidly accumulated in the nucleus by 15 minutes after treatment (Figure 3A). In order to further determine if the nuclear accumulation of annexin A2 induced by genotoxic agents was due to oxidative stress, we incubated TIME cells with the antioxidant agent N-acetyl cysteine (NAC) and then treated these cells with either H_2_O_2_ or IR. We observed that NAC blocked annexin A2 nuclear accumulation (Figure 3B, C), confirming that the nuclear accumulation of annexin A2 induced by genotoxic agents was due to oxidative stress. Next, we incubated TIME cells with different chromium isoforms that produce none (Cr^4+^), to intermediate levels (Cr^5+^) or very high levels (Cr^6+^) of oxidative stress and investigated the levels of nuclear annexin A2. We observed that hexavalent chromium (Cr^6+^) was the most potent inducer of nuclear annexin A2 accumulation (Figure 3D). These results support that annexin A2 accumulates in the nucleus in response to oxidative stress. We also examined the distribution of annexin A2 in response to oxidative stress by immunofluorescence microscopy. As shown in Figure 3E, oxidative stress resulting from the incubation of TIME cells with H_2_O_2_ caused the nuclear accumulation of annexin A2. Similar results were observed with MCF7 and A549 cells (Figure S1A, B).

### Nuclear Annexin A2 is not Associated with S100A10

**Figure 4:**
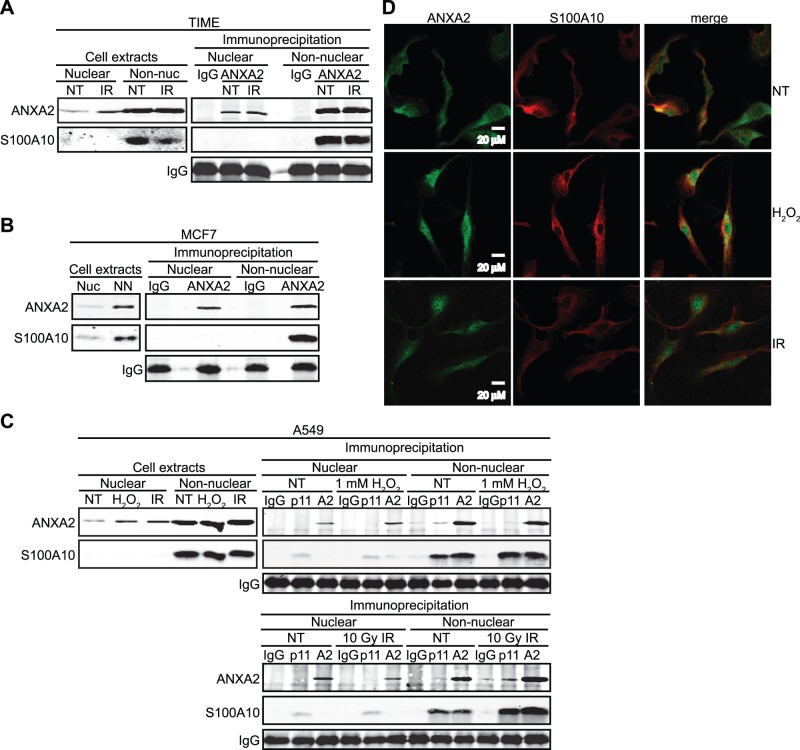
Nuclear annexin A2 is not associated with S100A10. (A) TIME cells were either not treated (NT) or treated with 10 Gy IR for 2 hours; (B) MCF7 cells were treated with 10 Gy IR for 2 hours; (C) A549 cells were either not treated (NT) or treated with 1 mM H_2_O_2_ or 10 Gy IR for 2 hours as indicated. (A–C) Nuclear and non-nuclear fractions were prepared followed by immunoprecipitation with the antibodies indicated. Nuclear and non-nuclear fractions (cell extracts) and immunoprecipitates were subjected to SDS-PAGE and analyzed by western blotting with the antibodies indicated. (D) TIME cells were either not treated (NT) or treated with 0.5 mM H_2_O_2_ for 30 minutes or 10 Gy IR for 1 hour. Cells were subjected to immunocytochemistry analysis with the antibodies indicated and visualized by confocal microscopy. Scale bar is 20 µM.

Annexin A2 exists in the cells mainly in two forms, as a monomer or in a heterotetrameric complex with its binding partner S100A10. We next investigated if S100A10 was involved in the nuclear accumulation of annexin A2 induced by genotoxic agents. TIME, A549 and MCF7 cells were either not treated or treated with gamma-radiation (IR) or H_2_O_2_. The nuclear and non-nuclear fractions were incubated with antibodies against annexin A2 or S100A10 and the immunoprecipitates were subjected to SDS-PAGE followed by western blot analysis (Figure 4). These results showed that S100A10 protein does not accumulate in the nucleus in response to either gamma-radiation or H_2_O_2_ and that the nuclear annexin A2 was not associated with S100A10 (Figure 4). As a control we demonstrated that non-nuclear annexin A2 readily co-immunoprecipitated with S100A10 (Figure 4). Immunofluorescence analysis further confirmed that S100A10 was not present in the nucleus in response to genotoxic agents (Figure 4D and S1). These results show that the annexin A2 that accumulates in the nucleus in response to genotoxic agents is not complexed with S100A10.

### The NES of Annexin A2 Regulates its Nuclear Accumulation in Response to Genotoxic Agents

**Figure 5:**
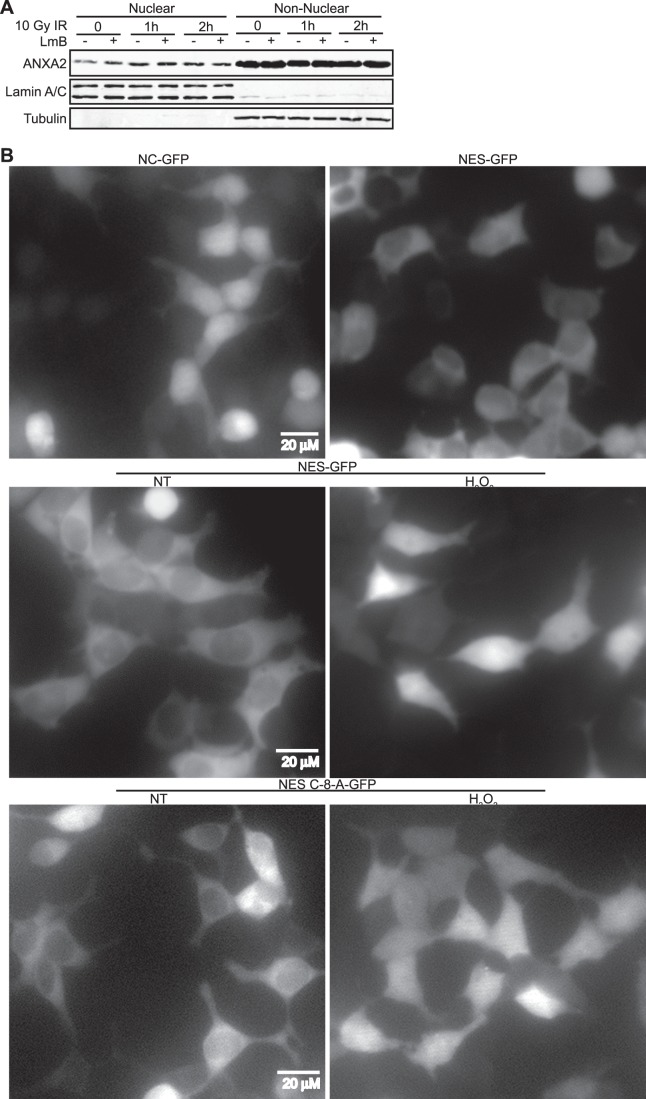
Annexin A2 nuclear accumulation is regulated by its NES. (A) TIME cells were treated with 10 Gy of IR in the absence or presence of 10 ng/ml Leptomycin B (LmB) for the times indicated. Nuclear and non-nuclear fractions were prepared. Identical ratios of the various protein fractions were subjected to SDS-PAGE followed by western blotting with the antibodies indicated. (B) 293T cells were transfected with Non-specific Control-GFP (NC-GFP), NES-GFP or NES-C-8-A-GFP constructs as indicated. 48 hours after transfection cells were either not treated (NT) or treated with H_2_O_2_ and the various GFP proteins were visualized by fluorescence microscopy. Scale bar is 20 µM.

Annexin A2 possesses a well described nuclear export sequence (NES) in the N-terminal region of the protein (^3^ **V**-X-X-X-**L**-X-X-**L**-X-**L** ^12^) [13]. Mutational analysis studies have shown that inactivation of the NES of annexin A2 results in the accumulation of the protein in the nucleus and that the CRM1 nuclear export inhibitor, leptomycin B (LmB) induces annexin A2 nuclear accumulation [13]. In order to investigate if the NES of annexin A2 was involved in the nuclear accumulation of annexin A2 induced by genotoxic agents, we incubated TIME cells with LmB and then treated these cells with IR. These data showed that treatment of cells with LmB or IR induced the nuclear accumulation of annexin A2 to similar levels and that treatment of cells with both IR and LmB did not further increase annexin A2 nuclear accumulation as compared to treatment with LmB or IR alone (Figure 5A). These results suggest that LmB and IR might induce the nuclear accumulation of annexin A2 by a common mechanism, namely through the inhibition of CRM1, since treatment of cells with both agents does not have an additive effect on annexin A2 nuclear accumulation. In order to determine if the NES of annexin A2 was sufficient to regulate the nuclear accumulation in response to genotoxic agents, we tagged the N-terminus of green fluorescent protein (GFP) either with the N-terminal 15 amino acids (^1^STVHEILCKLSLEGD^15^) of annexin A2 which contains the NES or with 15 random amino acids, as a non-specific control (NC) peptide. GFP was used in these experiments because it can be readily visualized by fluorescence microscopy and also because it is a relatively low molecular weight protein (27 kDa), like annexin A2 (36 kDa). We observed that GFP tagged with the control peptide (NC-GFP) was localized in the cytoplasm and in the nucleus of 293T cells, while GFP tagged with the NES of annexin A2 was excluded from the nucleus of these cells (Figure 5B (top panels), S3A and S3B). Interestingly, we observed that after treatment of the 293T cells with H_2_O_2_ the NES-GFP fusion protein was no longer excluded from the nucleus, its distribution was both nuclear and cytoplasmic, suggesting that oxidative stress either inactivates the NES or the nuclear transport mechanism responsible for excluding the NES-GFP protein from the nucleus (Figure 5B (middle right panel) and S3B). Next, we tagged GFP with a mutant NES where lysines 10 and 12 were replaced by alanine residues (NES-L-10/12-A-GFP) as these mutations inactivate the NES of annexin A2 [13]. We observed that the NES-L-10/12-A-GFP protein was localized in the nucleus and cytoplasm of 293T cells (Figure S4A), this result indicates that the NES of annexin A2 excludes GFP from the nucleus. As an extra control, we treated cells expressing NES-GFP with leptomycin B (LmB), which inhibits the nuclear export protein CRM1 and consequently leads to the nuclear accumulation of ANXA2. This result showed a nuclear and cytoplasmic distribution of the NES-GFP protein upon LmB treatment, as expected (Figure S4B).

Recent work from our laboratory has shown that annexin A2 has a redox sensitive cysteine, Cys-8, that is a target for oxidation by H_2_O_2_ [10]. Since Cys-8 is located in the NES it was reasonable to suspect that this residue might act as a redox sensor for the NES and inactivate the NES in response to oxidative stress. Therefore, we mutated this residue to alanine and expressed the NES-C-8-A-GFP construct in 293T cells. We reasoned that if the Cys-8 residue was involved in the inactivation of the NES by oxidative stress then its substitution with alanine would either block the ability of oxidative stress to inactivate the NES or this substitution could by itself inactivate the NES. We observed that Cys-8 mutation did not affect the nuclear exclusion of GFP in non-treated 293T cells and that NES-C-8-A-GFP accumulated in the nucleus in response to H_2_O_2_ treatment (Figure 5B (lower panels) and S4C). These data suggest that the Cys-8 of annexin A2 is not involved in the regulation of the NES of annexin A2 in response to oxidative stress.

### Annexin A2 Protects Cellular DNA from Damage

**Figure 6:**
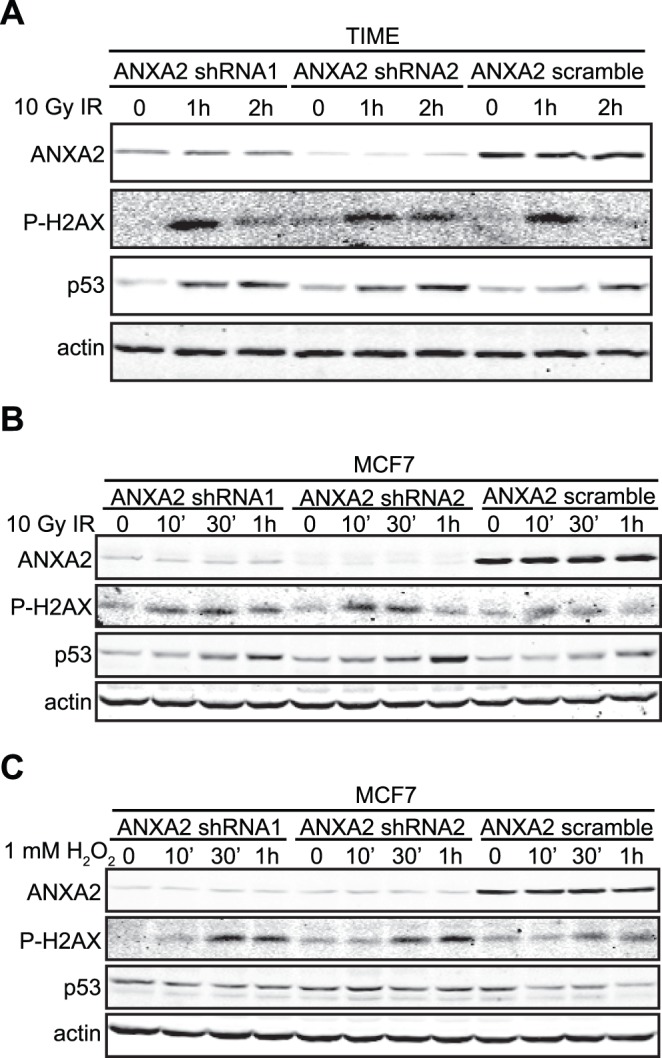
Annexin A2 protects cellular DNA from damage. (A) TIME ANXA2 shRNA1, TIME ANXA2 shRNA2 or TIME ANXA2 scramble cells were treated with 10 Gy IR for the times indicated; (B-C) MCF7 ANXA2 shRNA1, MCF7 ANXA2 shRNA2 or MCF7 ANXA2 scramble cells were treated with (B) 10 Gy IR for the times indicated or (C) 1 mM H_2_O_2_ for the times indicated. (A–C) 20 µg of each cell lysate was subjected to SDS-PAGE followed by western blot analysis with the antibodies indicated.

The observation that annexin A2, a redox regulatory protein [10], rapidly accumulates in the nucleus in response to genotoxic agents by a mechanism involving the production of ROS presented the possibility that annexin A2 might protect cellular DNA from oxidative damage. In order to investigate this hypothesis we established annexin A2 depleted and control cell lines and treated these cells with H_2_O_2_ or IR (Figure 6). DNA damage was measured using two DNA damage markers namely the phosphorylation of histone H2AX (P-H2AX) and the accumulation of p53 protein. The rapid phosphorylation of histone H2AX at serine 139 is a sensitive marker for DNA double-strand breaks induced by ionizing radiation or other genotoxic agents [31]. The tumor suppressor p53 protein accumulates in the nucleus upon DNA damage where it functions as a transcription factor regulating cell cycle arrest/apoptosis [32]–[34]. We observed that annexin A2 depleted cells showed increased H2AX phosphorylation and p53 accumulation upon IR treatment compared to control cells (Figure 6A, B). H_2_O_2_ treatment also increased H2AX phosphorylation but did not induce p53 accumulation during the time course analyzed (Figure 6C). Annexin A2 depleted cells showed increased H2AX phosphorylation upon H_2_O_2_ treatment compared to control cells (Figure 6C). However, annexin A2 depletion in both TIME and MCF7 cells did not by itself lead to a striking increase in DNA damage, as increased H2AX phosphorylation or accumulation of p53 in the non treated annexin A2 depleted cells was not observed. These results indicate that annexin A2 protects DNA from damage by genotoxic agents.

**Figure 7:**
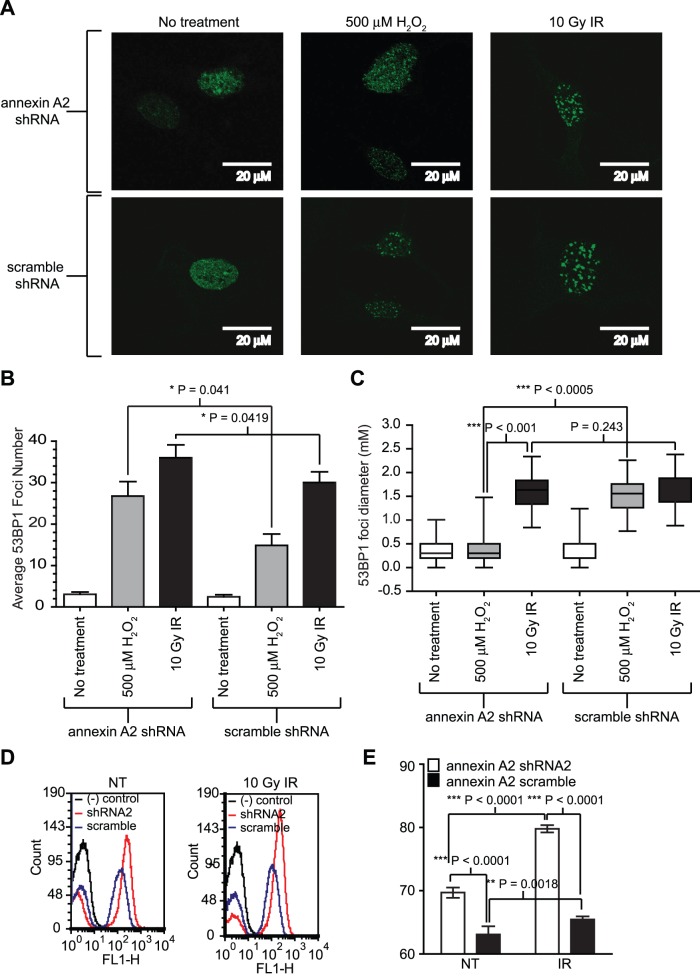
Annexin A2 depleted cells have higher number of 53BP1 foci and enhanced 8-oxy-G upon genotoxic stress compared to control cells. TIME annexin A2 shRNA2 and scramble (control) cells were plated on microscope cover slips and incubated at 37°C for 24 h. Cells were then treated with either 10 Gy IR or 500 µM H_2_O_2_ for 6 hours. Cells were permeabilized and incubated with anti-53BP1 antibody (SCBT, USA) for 1 hour. Subsequently, they were incubated with the appropriate Cy2-conjugated secondary antibody (Molecular Probes, USA) for 30 minutes. 53BP1 foci were visualized at 100× magnification using a Zeiss LSM 510 META - laser scanning confocal microscope. (A) Representative slides for 53BP1 foci staining for each cell line and treatment, as indicated. Scale bar is 20 µM. (B) Average number of 53BP1 foci for each cell line and treatment, as indicated. (C) 53BP1 foci diameter for each cell line and treatment, as indicated. (A–C) For each condition the number of 53BP1 foci was scored within 100 nuclei from triplicate cover slips. For each 53BP1 foci scored, the diameter was measured using the LSM imaging software Zen 2009 light edition. Statistical significance was assessed by two-way ANOVA or the two-tailed Students t-test. Statistical significance was defined as P<0.05. Results are expressed as the mean ± StDev. (D) TIME annexin A2 shRNA2 or control cells were either not treated or treated with 10 Gy IR for 6 hours. Cells were fixed, permeabilized and labeled with 8-oxy-binding-FITC conjugated protein (Argutus Medical), followed by FACS analysis. As a negative control we used TIME cells that were not incubated with 8-oxy-binding-FITC protein in order to establish the background fluorescence; (E) Percentage of 8-oxy-binding-FITC positively labeled cells from (D). Statistical significance was assessed by the two-tailed Students t-test, N = 6. Statistical significance was defined as *P<0.05, **P<0.002, *** P<0.0001. Results are expressed as the mean ± StDev.

The quantification of p53-binding protein 1 (53BP1) by fluorescence microscopy following genotoxic damage is an important method for the detection of DNA damage [35], [36]. This protein is recruited to nuclear foci which typically correspond to DNA strand breaks. We observed that both H_2_O_2_ and IR exposure triggered the accumulation of multiple 53BP1 foci (Figure 7A), consistent with the induction of DNA damage by these agents. We observed significantly higher numbers of 53BP1 foci in annexin A2 depleted cells treated with both H_2_O_2_ and IR compared to control cells (Figure 7B). However, even though IR produced a larger number of 53BP1 foci as expected, the most significant difference for the number of foci between annexin A2 depleted versus control cells was observed in cells treated with the reactive oxygen species, H_2_O_2_, suggesting that annexin A2 might play a role in protecting DNA from oxidative damage. Another interesting observation was that the 53BP1 foci formed in the ANXA2 depleted cells upon oxidative stress (H_2_O_2_ treatment) were significantly smaller than the foci size observed for the control cells (Figure 7C).

Our results suggested that annexin A2 might function in the nucleus to protect DNA from oxidative damage. Since IR can induce DNA damage/breaks in a ROS independent way, we further investigated if annexin A2 specifically protected DNA from IR induced oxidative damage. Genotoxic agents such as IR can cause oxidative damage to DNA which results in the formation of 7,8-dihydro-8-oxo-2′-deoxyguanine (8-oxo-G) base mutations in the DNA [37]. In order to investigate this possibility we treated annexin A2 depleted and control TIME cells with 10 Gy IR and measured oxidative DNA damage, by the formation of 8-oxo-G base mutations. We observed a slight increase in DNA damage in the non treated annexin A2 depleted cells, compared to control cells (Figure 7D, E). Interestingly, cells depleted of annexin A2 showed significantly elevated oxidative DNA damage after treatment with 10 Gy IR, compared to control cells (Figure 7D, E), further supporting a role for annexin A2 in protecting DNA specifically from oxidative damage.

## Discussion

In the current report we show that annexin A2 accumulates in the nucleus upon exposure of cells to different genotoxic agents and protects the cellular DNA from oxidative damage.

Our current studies show that the genotoxic agents: IR, UV-A, etoposide, Cr^6+^ and H_2_O_2_ induce the accumulation of annexin A2 in the nucleus. We used different genotoxic agents that induce DNA damage in different ways in order to show that ANXA2 accumulates in the nucleus in response to the generation of ROS induced by these genotoxic agents and not in response to DNA damage/signaling, to further support this result we showed that NAC treatment inhibits the nuclear accumulation of ANXA2 that is induced by the genotoxic agents. In summary, the nuclear accumulation of ANXA2 seems to occur in response to different types of DNA damage and what is common to these genotoxic damages is the production of ROS which regulates ANXA2 nuclear accumulation. This result led us to hypothesize that it is the redox function of ANXA2 that most likely plays a role in DNA protection, since the mechanism(s) of damage and repair for the different genotoxic damages investigated are distinct. Since ANXA2 interacts directly with H_2_O_2_ and plays a significant role in the regulation of ROS levels during oxidative stress [10], we speculate that a probable mechanism by which ANXA2 protects DNA from damage is through the regulation of the ROS produced by the genotoxic agents. We have previously shown that ANXA2 knockdown cells are more sensitive to death induced by several chemotherapeutic agents [10]. This result suggested that the ANXA2 knockdown cells treated with the same dosage of ROS inducing chemotherapeutic agents accumulate more ROS in comparison to control cells, leading to enhanced death of the ANXA2 knockdown cells.

Other annexins have been shown to act as redox proteins. In contrast to the Cysteine-8 residue of ANXA2, a putative redox-active cluster is exhibited by many plant annexins. The cluster is formed by two adjacent cysteine residues and the sulphur of a nearby methionine residue and is located in helices IIB and IIIE [38]. Annexin A1 from Arabidopsis thaliana (AnnAt1) participates in modulating the excessive levels of reactive oxygen species during oxidative burst in plants [39]. AnnAt1 from *A. thaliana*, expressed in *Escherichia coli* and *Nicotiana benthamiana* possesses peroxidase activity [40]. Interestingly, hydrogen peroxide accumulation in guard cells was reduced in plants over-expressing AnnAt1 and increased in knockout plants [41]. Plant AnnAt1 as most annexins, does not possess a nuclear localization sequence; however, translocation of the protein to the nucleus has been observed upon stress stimulation [39], [42]–[44]. Similarly, treatment of cells with H_2_O_2_ has been shown to cause the translocation of mammalian annexin I from the cytoplasm to the nucleus [40]. Therefore ANXA2 is not the only member of the annexin family to act as a redox protein and demonstrate redox-dependent movement to the nucleus.

Typically, proteins that shuttle between the nucleus and cytoplasm have a nuclear localization signal (NLS) sequence and a NES (reviewed in [45]–[47]). Transport of a protein into the nucleus is initiated with its binding to importin α via its NLS sequence which then binds to importin β to form a ternary complex. The complex is then transferred to the internal face of the nuclear pore, recognized by the nuclear pore complex (NPC) and transported further into the nucleoplasm. The NES of the nuclear protein then binds to CRM1 (exportin1) and the resulting complex is then exported from the nucleus. Although the mechanism by which annexin A2 enters the nucleus is not thought to involve a NLS, the transport of annexin A2 from the nucleoplasm to the cytoplasm is regulated by its NES. NES sequences are short sequence motifs which are necessary and sufficient to mediate the nuclear export of large carrier proteins. Important for their function is a characteristic spacing of hydrophobic residues, mainly leucine or isoleucine. NES typically consist of a sequence of hydrophobic amino acids which follow the pattern L-X^(1–4)^-L-X^(2)^-L-^(X)^-L, where L is usually a hydrophobic residue [46], [48], [49]. A nuclear export signal sequence (^3^ **V**HEI**L**CK**L**S**L**E^13^) has been identified in annexin A2 by Creutz’s group [13]. This group observed that the nuclear export of annexin A2 was inhibited by leptomycin B (LmB). Since LmB inactivates CRM1, it was suggested that annexin A2 was exported from the nucleus by the CRM1 pathway. Thus this group suggested that annexin A2 enters the nucleus by an unknown mechanism but is prevented from accumulating in the nucleus by the dominance of the NES. In the presence of genotoxic agents we observed that annexin A2 accumulated in the nucleus suggesting that genotoxic agents stimulate the nuclear influx of annexin A2, inhibit its export from the nucleoplasm or influence both processes. It was therefore interesting that the GFP fusion protein consisting of the annexin A2 NES fused to the N-terminus of GFP, accumulated in the nucleus in response to H_2_O_2_. This result suggested that the nuclear accumulation of annexin A2 can be regulated by its NES. Since the nuclear accumulation of annexin A2 was blocked by the antioxidant, NAC and stimulated by H_2_O_2_, we suspected that oxidation of Cys-8 within the NES might inactivate the NES and prevent the export of the nuclear protein. However, the GFP fusion protein, consisting of a Cys-8-Ala mutation in the NES was excluded from the nucleus in non treated cells and also resulted in GFP accumulation in the nucleus in response to H_2_O_2_. This result suggests that the Cys-8 residue of annexin A2 is not involved in the regulation of its NES. Since the CRM1 dependent export of nuclear proteins can be inhibited by oxidative stress [50], we hypothesized that oxidative stress inactivates the exportin CRM1 resulting in the inhibition of the export of annexin A2 from the nucleus and consequent nuclear accumulation of the protein.

The N-terminal region of annexin A2 not only contains a NES but also contains the binding site for S100A10. The key residues for S100A10 binding are Val-3, Ile-6, Leu-7, and Leu-10 [51]–[53]. Considering that these residues are also part of the NES, it was possible that genotoxic agents and/or oxidative stress might promote the binding of S100A10 to annexin A2 which would be predicted to block the NES and allow nuclear accumulation of the annexin A2/S100A10 complex. However, we observed that annexin A2 but not its binding partner S100A10 accumulated in the nucleus in response to genotoxic agents. This result is in accordance with another study that reported that monomeric annexin A2 accumulated in the nucleus in response to LmB treatment [13]. In the present study we show that annexin A2 that accumulates in the nucleus in response to genotoxic agents is also a monomer.

Genotoxic agents are known to cause DNA damage by interacting with DNA and causing DNA strand breaks. The rapid phosphorylation of histone H2AX at serine 139 is a sensitive marker for DNA double-strand breaks induced by IR or other genotoxic agents [31]. Interestingly, we observed that treatment of cells with IR or H_2_O_2_ resulted in significantly higher levels of P-H2AX if the cells were depleted of annexin A2. Several proteins involved in DNA repair and DNA damage signaling such as phosphorylated histone 2A family member X (γ-H2AX) and the tumour suppressor p53 binding protein 1 (53BP1) have been shown to produce discrete foci that co-localize to DNA breaks [35], [54]. Our data also showed that annexin A2 depleted cells formed significantly more 53BP1 foci after H_2_O_2_ and IR exposure compared to control cells. These results indicate that annexin A2 plays a role in protecting DNA from genotoxic damage. It was interesting to observe that the foci formed in the annexin A2 depleted cells upon treatment with H_2_O_2_ were significantly smaller compared to control cells. This result suggests that annexin A2 might be involved in the formation of DNA repair foci upon oxidative stress. Taking into account annexin A2 already known functions, we speculate that annexin A2 might function as a scaffold protein and/or a redox regulatory protein, within the DNA repair foci. These function(s) could explain why the foci formed in the annexin A2 depleted cells upon H_2_O_2_ treatment are significantly smaller compared to control cells.

Many genotoxic agents are known to cause DNA damage not only by interacting directly with DNA and causing DNA strand breaks, but also indirectly by a mechanism that involves the production of ROS/oxidative stress [55]. The question therefore was if the role of annexin A2 as a cellular redox regulatory protein [10] was relevant to its ability to protect cells from DNA damage. Our observation that annexin A2 depletion resulted in cells that were more sensitive to DNA damage induced by H_2_O_2_ suggested that annexin A2 might mitigate oxidative stress and by this mechanism prevent DNA damage. Genotoxic agents such as IR can cause oxidative damage to DNA which result in the formation of 8-oxo-G base mutations in the DNA [37]. Interestingly, we observed that in response to IR, cells depleted of annexin A2 showed more oxidative DNA damage than control cells, as measured by the presence of 8-oxo-G. This result supports that annexin A2 redox regulatory function is important for its ability to protect cellular DNA from oxidative damage.

In summary, this is the first report that shows annexin A2 nuclear accumulation in response to genotoxic agents and its role in mitigating DNA damage.

## Supporting Information

Figure S1 **Annexin A2 monomer accumulates in the nucleus in response to oxidative and genotoxic stresses.** (A) MCF7 cells or (B) A549 cells were either not treated (NT) or treated with 0.5 mM H_2_O_2_ for 30 minutes or 10 Gy IR for 1 hour as indicated. Cells were fixed with 2% formaldehyde, permeabilized with 0.1% Triton X-100, blocked with IgG and incubated with antibodies against annexin A2, hnRNP A2/B1 or S100A10 as indicated. Cells were visualized by confocal microscopy. Scale bar is 20 µM.
(EPS)

Figure S2 **Annexin A2 accumulates in the nucleus in response to genotoxic stress.** (A) HUVEC, (B) A549, (C) MCF7 or (D) HCT 116 cells were treated with 10 Gy of gamma radiation (ionizing radiation- IR) for the times indicated. Nuclear and non-nuclear fractions were prepared. Identical ratios of nuclear and non-nuclear lysates were subjected to SDS-PAGE followed by western blot analysis with the antibodies indicated. Protein markers for the nuclear fraction include nucleolin and lamin A/C and the protein marker for the cytoplasmic fraction is tubulin. Cell culture of HCT 116 and HUVEC cells is described in File S1.
(EPS)

Figure S3 **The NES of annexin A2 is inactivated upon oxidative stress.** 293T cells were transfected with (A) Non-specific Control-GFP (NC-GFP) or (B) NES-GFP constructs, as indicated. 48 hours after transfection cells were either not treated (NT) or treated with H_2_O_2_ as indicated and the various GFP proteins were visualized by fluorescence microscopy. Scale bar is 20 µM. Plasmids are detailed in File S1.
(EPS)

Figure S4 **Characterization of the NES of annexin A2.** 293T cells were transfected with (A) NES-L-10/12-A-GFP, (B) NES-GFP or (C) NES-C-8-A-GFP constructs as indicated. 48 hours after transfection cells were either not treated (NT); treated with leptomycin B (LmB) or treated with H_2_O_2_ as indicated and the various GFP proteins were visualized by fluorescence microscopy. Scale bar is 20 µM. Plasmids are detailed in File S1.
(EPS)

File S1 (DOC)
