# Supplementary material for: Genotoxic Agents Promote the Nuclear Accumulation of Annexin A2: Role of Annexin A2 in Mitigating DNA Damage
Source: PLoS One. 2012 Nov 30;7(11):e50591. doi: 10.1371/journal.pone.0050591 (PMC3511559; doi:10.1371/journal.pone.0050591)
Supplement: File S1 — (DOC) [file pone.0050591.s005.doc]

Genotoxic agents promote the nuclear accumulation of annexin A2: Role of annexin A2 in mitigating DNA damage.

**Patricia A. Madureira^1, 3^, Richard Hill^2^, Patrick W. K. Lee^2^ and David M. Waisman^1^**

**Inventory of supporting information:**

Supporting information includes supporting materials and methods and four supporting figures.

**SUPPORTING MATERIALS AND METHODS**

Cell Culture, transfections and cell lines

HCT 116 cells were obtained from ATCC and maintained in Dulbecco's modified Eagle's medium (Invitrogen) supplemented with 10% fetal bovine serum (FBS) and 100 U/ml of penicillin/streptomycin, in a humidified incubator in an atmosphere of 5% CO_2_ at 37°C. HUVEC cells were isolated from human umbilical cords and maintained in MCDB 131 medium supplemented with 10% FBS and 100 U/ml of penicillin/streptomycin, in a humidified incubator in an atmosphere of 5% CO_2_ at 37^0^C.

Plasmids

pNES-GFP was constructed by cloning the double stranded oligo: 5’-GA TCT ATG TCT ACT GTT CAC GAA ATC CTG TGC AAG CTC AGC TTG GAG GGT GAT T-3’ and 5’-CT AGA ATC ACC CTC CAA GCT GAG CTT GCA CAG GAT TTC GTG AAC AGT AGA CAT A-3’ into the pEGFP-C1 plasmid (BD Biosciences Clontech); pNES C-8-A-GFP was constructed by cloning the double stranded oligo: 5’-GA TCT ATG TCT ACT GTT CAC GAA ATC CTG GCA AAG CTC AGC TTG GAG GGT GAT T-3’ and 5’-CT AGA ATC ACC CTC CAA GCT GAG CTT TGC CAG GAT TTC GTG AAC AGT AGA CAT A-3’ into the pEGFP-C1 plasmid (BD Biosciences Clontech); pNES L-10/12-A-GFP was constructed by cloning the double stranded oligo: 5’-GA TCT ATG TCT ACT GTT CAC GAA ATC CTG TGC AAG GCG AGC GCG GAG GGT GAT T-3’ and 5’-CT AGA ATC ACC CTC CGC GCT CGC CTT GCA CAG GAT TTC GTG AAC AGT AGA CAT A-3’ into the pEGFP-C1 plasmid (BD Biosciences Clontech); pNC-GFP was constructed by cloning the double stranded oligo: 5’-GA TCT ATG GAC TCT CTC ATT GAG ATC ATC TGC TCC AGA ACC AAC CAG GAG CTG T-3’ and 5’-CT AGA CAG CTC CTG GTT GGT TCT GGA GCA GAT GAT CTC AAT GAG AGA GTC CAT A-3’ into the pEGFP-C1 plasmid (BD Biosciences Clontech). pSUPER-retro-ANXA2 shRNA1 was constructed by cloning the dsDNA oligo 5'-GAT CCC CCC TGG TTC AGT GCA TTC AGT TCA AGA GAC TGA ATG CAC TGA ACC AGG TTT TTA-3' and 5'-AGC TTA AAA ACC TGG TTC AGT GCA TTC AGT CTC TTG AAC TGA ATG CAC TGA ACC AGG GGG-3' into pSUPER.retro.puro (OligoEngine), pSUPER-retro-ANXA2 shRNA2 was constructed by cloning the dsDNA oligo 5'-GAT CCC CGT GCA TAT GGG TCT GTC AAT TCA AGA GAT TGA CAG ACC CAT ATG CAC TTT TTA-3' and 5'-AGC TTA AAA AGT GCA TAT GGG TCT GTC AAT CTC TTG AAT TGA CAG ACC CAT ATG CAC GGG-3' into pSUPER.retro.puro (OligoEngine) and the pSUPER-retro-ANXA2 scramble was constructed by cloning the dsDNA oligo 5'-GAT CCC CGT GCA TAT GGG TCT GTC CAT TAG AGA GAT TGA CAG ACC CAT ATG CAC TTT TTA-3' and 5'-AGC TTA AAA AGT GCA TAT GGG TCT GTC AAT CTC TCT AAT GGA CAG ACC CAT ATG CAC GGG-3' into pSUPER.retro.puro (OligoEngine).
